# Add-on anticholinergic therapy for residual nocturia in patients with lower urinary tract symptoms receiving α1-blocker treatment: a multi-centre, prospective, randomised study

**DOI:** 10.1007/s00345-014-1399-x

**Published:** 2014-09-16

**Authors:** Osamu Yokoyama, Akira Tsujimura, Hironobu Akino, Naoki Segawa, Satoshi Tamada, Naoki Oguchi, Yasuhide Kitagawa, Hidenori Tsuji, Akihiko Watanabe, Teruo Inamoto, Nobutaka Shimizu, Yasuyoshi Fujiuchi, Yoji Katsuoka, Haruhito Azuma, Tadashi Matsuda, Mikio Namiki, Hirotsugu Uemura, Akihiko Okuyama, Norio Nonomura, Hideki Fuse, Tatsuya Nakatani

**Affiliations:** 1 Department of Urology, Faculty of Medical Science, University of Fukui, Fukui, Japan; 2 Department of Urology, Osaka University Graduate School of Medicine, Suita, Japan; 3 Department of Urology, Osaka Medical College, Osaka, Japan; 4 Department of Urology, Osaka City University, Osaka, Japan; 5 Department of Urology and Andrology, Kansai Medical University, Osaka, Japan; 6 Department of Integrative Cancer Therapy and Urology, Kanazawa University Graduate School of Medical Science, Kanazawa, Japan; 7 Department of Urology, Kinki University School of Medicine, Osaka, Japan; 8 Department of Urology, Graduate School of Medicine and Pharmaceutical Sciences for Research, University of Toyama, Toyama, Japan

**Keywords:** Anticholinergic, Nocturia, Nocturnal urine volume, Overactive bladder, Benign prostatic hyperplasia, Imidafenacin

## Abstract

**Purpose:**

To evaluate the efficacy and safety of imidafenacin (IM), a novel short half-life anticholinergic, as add-on therapy for male LUTS with nocturia and nocturnal polyuria.

**Materials and methods:**

This multicenter, prospective, randomized, open-labelled study was conducted and involved men who had frequency, urgency, and nocturia despite receiving a stable dose of α1-blocker for ≥1 month. Subjects were randomised to control (α1-blocker alone), IM twice/day (α1-blocker +0.1 mg imidafenacin twice daily), or IM nightly (α1-blocker plus 0.1 mg imidafenacin nightly) group; the treatment period was 8 weeks. Primary endpoints included improvements in night-time frequency and Nocturia Quality of Life Questionnaire (N-QOL) scores. Secondary endpoints included changes from the baseline in frequency volume chart variables, and post-void residual volume.

**Results and limitations:**

Compared with the controls, IM twice/day and IM nightly patients had a significantly lower night-time frequency (changes from baseline: 0.1 ± 0.8 in control, −0.6 ± 0.9 in IM twice/day, and −0.4 ± 1.0 in IM nightly, *p* = 0.5227, 0.0006 and 0.0143, respectively). The hours of undisturbed sleep and N-QOL score were significantly improved in IM twice/day group, though not IM nightly group. Nocturnal urine volume was significantly reduced in IM nightly group, although total urine volume remained unchanged.

**Conclusions:**

A short half-life anticholinergic is suggested to be safe and effective as an add-on therapy for residual nocturia in patients with male LUTS receiving α1-blocker treatment. Anticholinergic administration nightly could reduce the nocturnal urine volume.

## Introduction

Nocturia (night-time voiding)—defined as ‘the need to get up once or more times for nocturnal voids’ [1]—is the most common lower urinary tract symptom (LUTS), reportedly observed in up to 69 % of cases [2]. Since its prevalence increases with age [2, 3], it is more common in the elderly, and it greatly affects general health and quality of life [4–6]. Many individuals with nocturia, particularly elderly men, have other LUTS, including urinary frequency, weak stream, and urgency. In men, these symptoms are primarily attributed to benign prostatic hyperplasia (BPH). Therapy typically involves treatment for detrusor overactivity or bladder outlet obstruction. However, while either or both these aetiologies may underlie nocturia, therapy may fail because of an often overlooked component, namely nocturnal polyuria (NP) [7].

Treatment for voiding and/or storage symptoms suggestive of BPH is now the initial choice for nocturia, and α_1_-blockers, which target the dynamic component of prostatic obstruction, remain the most widely used pharmacological agents [8]. α_1_-Blockers for LUTS suggestive of BPH are expected to alleviate storage symptoms including nocturia. However, they are not adequately effective for nocturia-related sleep disturbance. Anticholinergics with α_1_-blockers have shown statistical success in some groups, but the clinical significance of their effects is limited [9]. Anticholinergics reportedly improve overactive bladder (OAB) in nocturia by increasing bladder capacity but have no effect on NP [10]. We previously reported that imidafenacin decreased urine volume via suppression of C-fibres in the rat bladder [11] and clinically improved nocturia and reduced nocturnal urine volume [12]. However, the latter study was conducted mainly for female OAB patients. Thus, it remains unclear whether anticholinergics are effective for treating nocturia and NP, especially among men. Studies have reported that anticholinergics with α_1_-blockers are effective and safe for male LUTS [13–15]. However, these studies did not focus on nocturia and NP evaluated using urinary frequency volume charts (FVCs). Only limited data support the use of α_1_-blocker and anticholinergics; moreover, nocturia has rarely been used as a primary endpoint when studying these drug classes, and such studies have not consistently controlled for the effect of NP [16].

In this study, we investigated whether imidafenacin, a novel anticholinergic that has been marketed in Japan since 2007 [17, 18], is effective for treating nocturia and NP.

## Materials and methods

This multi-centre, randomised, 8-week study was conducted at eight university hospitals in Japan between August 2009 and March 2011. The study, named the Good-Night study, complied with the International Conference on Harmonization Good Clinical Practice Guideline and the Declaration of Helsinki. Further, the study protocol was approved by the review board of each hospital, and all subjects provided written informed consent. The clinical study design is posted at http://www.umin.ac.jp/ctr/ (Unique ID: UMIN000002344).

### Subject selection

Before the subjects were enrolled in this study, they underwent behavioural therapy, including lifestyle guidance. Eligible subjects included men with persistent nocturia (≥2 voids/night) and LUTS including OAB symptoms [mean urinary frequency ≥8 times/24 h and ≥1 micturition-related urgency episode/week, evaluated using the Overactive Bladder Symptom Score (OABSS) [19]] who had been receiving a stable dose of an α_1_-blocker for ≥1 month. The International Prostate Symptom Score (IPSS)/IPSS-QOL was also evaluated. The exclusion criteria were as follows: post-void residual volume (PVR) ≥50 mL, complications that are contraindications for anticholinergics, high possibility of prostate and bladder cancer, acute active urinary tract infection, indwelling or intermittent urethral catheterisation, comorbidities that affect nocturia (sleep apnoea syndrome, restless leg syndrome, insomnia, etc.), hormone or 5-α-reductase inhibitor therapy that was started within the past 6 month, shift work and circadian rhythm disorder, irregular lifestyle, electrostimulation therapy or bladder training in the 10 days before the run-in period, contraindication for imidafenacin (primary angle-closure glaucoma, urinary retention, obstructive intestinal disease, paralytic ileus, gastrointestinal atony, and myasthenia gravis), and ineligibility as judged by the investigator in charge.

### Study design

Eligible subjects were randomised to receive an 8-week continuous treatment with an α_1_-blocker alone (control), α_1_-blocker +0.1 mg imidafenacin twice daily (IM twice/day), or α_1_-blocker plus 0.1 mg imidafenacin nightly (IM nightly). The subjects were randomly assigned to the three groups in a random sequence. Random assignment was performed by the central registration system of an independent organisation, and age was used as a factor in the assignment. The α_1_-blockers were selected from common ones used in Japan (tamsulosin, naftopidil, and silodosin).

### Clinical assessments

The primary efficacy endpoints were improvements in night-time frequency as determined by the FVC, which was a 3-day bladder diary, and the Nocturia Quality of Life Questionnaire (N-QOL) score [20]. The N-QOL includes two domains, namely Sleep/Energy and Bother/Concern, and the total score and domain scores were calculated on a scale of 0–100, with 100 indicating ideal conditions. Secondary endpoints were other FVC variables, including hours of undisturbed sleep (HUS), 24-h micturition frequency, daytime micturition frequency, nocturnal/daytime urine volume, nocturnal polyuria index (NPi; defined as nocturnal urine volume/24-h urine output), urine volume voided/void, PVR, OABSS, IPSS, IPSS-QOL, and at baseline and at weeks 4 and 8. NP was diagnosed if the NPi was ≥33 %. Prostate volume and PVR were measured by ultrasound.

Adverse events (AEs) were classified by the investigator according to severity and relationship to the treatment.

### Statistical analysis

Differences in FVC variables between treatment groups at baseline were evaluated using analysis of variance (ANOVA) at a significance level of 5 %; the N-QOL score and OABSS were evaluated using the two-sided Kruskal–Wallis test. Intra- and intergroup numerical changes from baseline to 4 and 8 weeks were evaluated using paired and unpaired *t* tests, respectively (control vs. IM twice/day or IM nightly). Intra- and intergroup changes in categorical variables, including the N-QOL score and OABSS, from baseline to 4 and 8 weeks were evaluated using the Wilcoxon and Mann–Whitney *U* tests, respectively (control vs. IM twice/day or IM nightly). The distribution of α_1_-blocker use during treatment and the mean duration of α_1_-blocker use before study entry were evaluated using Fisher’s exact test. Assessments of safety and tolerability were based on the findings for all patients who received at least one dose of the study medication. A two-sided significance level of 5 % was applied for all statistical tests, except those conducted on baseline characteristics. All analyses were performed using SAS 9.1 (SAS Institute Inc., Cary, NC, USA).

## Results

### Subject population

**Fig. 1:**
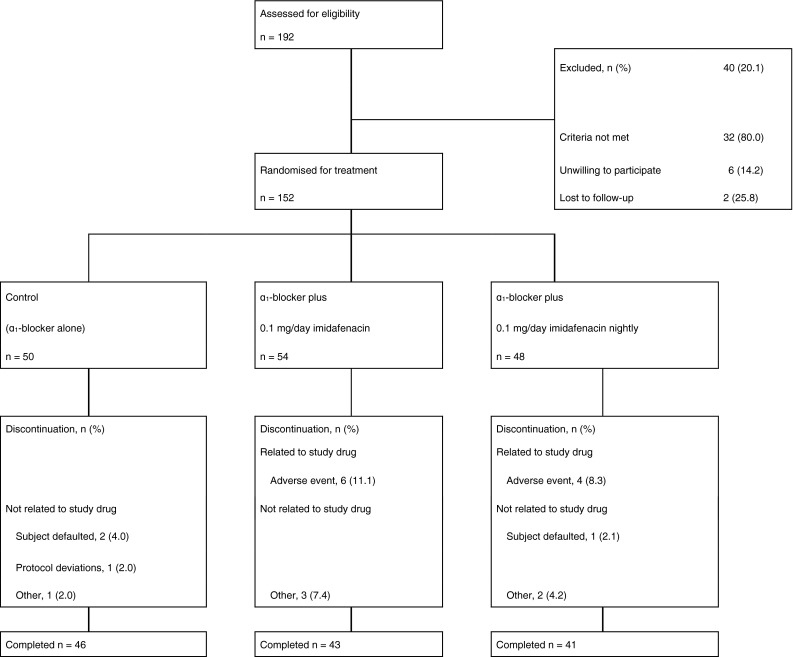
Disposition of subjects assigned to the study treatments

Patient disposition is summarised in Fig. 1. The baseline demographic and clinical characteristics were similar among the three treatment groups (Table 1). From the FVCs, 78 % (101/130) of the subjects were found to have NP. The distribution of α_1_-blocker use did not differ significantly among the three groups (Table 2), and 75 % (98/130) of the subjects used an α_1_-blocker for ≥3 month.

**Table 1:** Patient demographics and background

	Control (*n* = 46)	IM twice/day (*n* = 43)	IM nightly (*n* = 41)	*p* value
*Demographics*				
Age (years)	73.3 ± 6.7	74.7 ± 6.2	74.7 ± 7.6	0.5376^†^
BMI (kg/m^2^)	23.1 ± 3.2	23.1 ± 4.0	22.8 ± 3.0	0.8956^†^
Prostate volume (mL)	31.7 ± 12.6	28.1 ± 11.3	31.4 ± 15.6	0.4437^†^
PSA (ng/mL)	2.6 ± 2.2	2.3 ± 2.5	2.4 ± 2.0	0.7707^†^
*Comorbidity (%)*				
Hypertension	28.3	25.6	26.8	0.9689^‡^
Diabetes	4.3	14.0	14.6	0.2070^‡^
Renal disease	0.0	0.0	2.4	0.3154^‡^
Cerebrospinal disease	6.5	7.0	12.2	0.6750^‡^
PVR (mL)	18.9 ± 16.8	15.9 ± 13.5	23.4 ± 14.8	0.7978^†^
OABSS	7.84 ± 2.3	7.67 ± 2.3	7.79 ± 2.4	0.8612^†^
IPSS	15.5 ± 7.7	14.6 ± 7.5	15.4 ± 6.8	0.7927^†^
IPSS-QOL	4.24 ± 1.04	4.4 ± 1.16	4.25 ± 1.24	0.7915^†^

All values were expressed as mean ± SD

*BMI* body mass index, *PSA* prostate-specific antigen, *PVR* post-void residual volume, *OABSS* Overactive Bladder Symptom Score, *IPSS* International Prostate Symptom Score, *ANOVA* analysis of variance

Control, α-blocker alone; IM twice/day, α-blocker plus 0.1 mg imidafenacin twice daily; IM nightly, α-blocker plus 0.1 mg imidafenacin nightly

^†^ *p* value: intergroup (control vs. 0.1 mg imidafenacin twice/day or 0.1 mg imidafenacin nightly); ANOVA

^‡^ *p* value: intergroup (control vs. 0.1 mg imidafenacin twice/day or 0.1 mg imidafenacin nightly); Fisher’s exact test

**Table 2:** α-Blocker use according to the enrolled subjects

	Control (*n* = 46)	IM twice/day (*n* = 43)	IM nightly (*n* = 41)	*p* value^†^
α-Blocker [*n* (%)]				
Tamsulosin	13 (28.3 %)	9 (20.9 %)	15 (36.6 %)	0.6373
Naftopidil	18 (39.1 %)	19 (44.2 %)	15 (36.6 %)	
Silodosin	15 (32.6 %)	15 (34.9 %)	11 (26.8 %)	
Duration of α-blocker use before study entry [*n* (%)]				
≥1 month	6 (13.0 %)	8 (18.6 %)	7 (17.1 %)	0.6887
≥2 months	4 (8.7 %)	5 (11.6 %)	2 (4.9 %)	
≥3 months	6 (13.0 %)	5 (11.6 %)	2 (4.9 %)	
≥6 months	30 (65.2 %)	25 (58.1 %)	30(73.2 %)	

All values were expressed as mean ± SD

Control, α-blocker alone; IM twice/day, α-blocker plus 0.1 mg imidafenacin twice daily; IM nightly, α-blocker plus 0.1 mg imidafenacin nightly

^†^ *p* value: intergroup (control vs. 0.1 mg imidafenacin twice/day or 0.1 mg imidafenacin nightly); Fisher’s exact test

### Frequency volume charts

Compared with the control group, those treated with imidafenacin showed a significantly greater reduction in the nocturnal micturition (Table 3). Daytime frequency improved in the IM twice/day group but not in the IM nightly group (Table 3). The nocturnal urine volume in the control group remained unchanged throughout the study period, but imidafenacin treatment tended to reduce nocturnal urine production; in particular, 0.1 mg imidafenacin administered nightly reduced urine production significantly compared with that in the control group (Table 3). Further, in the IM nightly group, the 24-h urine volume remained unchanged, but NPi reduced significantly from the baseline. HUS and urine volume voided/void at night significantly improved in the IM twice/day group but not in the IM nightly group.

**Table 3:** Effect of treatment on frequency volume chart variables and N-QOL

	Control	IM twice/day	IM nightly
*Baseline parameter of frequency volume chart and N-QOL*			
24-h frequency	11.0 ± 0.5	10.9 ± 0.4	11.7 ± 0.3
Daytime frequency	8.6 ± 0.4	7.8 ± 0.3	8.7 ± 0.3
Nocturnal frequency	2.4 ± 0.2	3.2 ± 0.3	3.1 ± 0.2
24-h urine volume (mL)	1,807.2 ± 114.4	1,705.6 ± 88.3	1,731.0 ± 89.3
Daytime urine volume (mL)	1,064.6 ± 84.2	919.5 ± 60.5	1,006.7 ± 59.9
Nocturnal urine volume (mL)	740.9 ± 54.5	786.9 ± 50.8	717.3 ± 45.6
NPi (%)	42.1 ± 2.1	46.5 ± 1.9	41.6 ± 1.5
HUS (min)	173.7 ± 13.2	152.6 ± 11.2	153.5 ± 10.4
Urine volume voided/void (mL)	171.8 ± 11.0	161.7 ± 8.3	150.1 ± 7.7
Urine volume voided/void in the daytime (mL)	125.3 ± 8.7	117.1 ± 6.1	117.6 ± 6.5
Urine volume voided/void at night (mL)	372.5 ± 29.0	313.4 ± 26.8	256.4 ± 16.1
Total score	67.41 ± 2.91	66.24 ± 3.05	67.71 ± 3.14
Sleep/energy	70.63 ± 2.98	73.08 ± 2.95	71.05 ± 3.43
Bother/concern	64.19 ± 3.24	59.40 ± 3.64	64.36 ± 3.31

	Weeks (vs. baseline)		*p* value (vs. baseline)^†^		*p* value (vs. control)^†^	*p* value (vs. baseline)^‡^		*p* value (vs. control)^†^	*p* value^‡^
*Changes from baseline in the frequency volume chart and N*-*QOL*									
Primary endpoints									
Nocturnal frequency	4	0.19 ± 0.15		−0.49 ± 0.12	0.0003	0.0007	−0.32 ± 0.15	0.0331	0.0156
	8	0.08 ± 0.12		−0.57 ± 0.15	0.0006	0.0014	−0.41 ± 0.16	0.0143	0.0175
Total score of N-QOL	4	3.52 ± 1.85	0.0472	5.72 ± 1.87	0.0004		6.03 ± 2.25	0.0071	
	8	4.51 ± 2.19	0.0146	11.22 ± 2.36	<0.0001	0.0113	8.99 ± 2.13	<0.0001	
N-QOL subscale: Sleep/Energy	4	2.98 ± 1.79		3.95 ± 2.34	0.0419		4.06 ± 2.27		
	8	2.98 ± 1.99		7.26 ± 2.42	0.001		8.33 ± 2.46	0.0011	
N-QOL subscale: Bother/Concern	4	4.07 ± 2.3		7.48 ± 1.99	0.0003		8.00 ± 2.75	0.0022	
	8	6.05 ± 2.7		15.17 ± 3.11	<0.0001	0.0268	9.65 ± 2.41	0.0002	
Secondary endpoints									
24-h frequency	4	−0.18 ± 0.3		−0.83 ± 0.28	0.005		−1.02 ± 0.32	0.0026	
	8	0.43 ± 0.33		−1.18 ± 0.3	0.0004	0.0007	−0.82 ± 0.31	0.0105	0.0072
Daytime frequency	4	−0.36 ± 0.24		−0.35 ± 0.24			−0.7 ± 0.24	0.0064	
	8	0.29 ± 0.28		−0.67 ± 0.25	0.0113	0.013	−0.4 ± 0.22		
Daytime urine volume (mL)	4	−2.6 ± 44.7		80.7 ± 33.8	0.0222		−30.9 ± 40.1		
	8	79.6 ± 52.4		27.1 ± 44.4			−5.0 ± 45.8		
Nocturnal urine volume (mL)	4	14.5 ± 26.6		−37.8 ± 30.2			−49.7 ± 40.0		
	8	15.7 ± 26.1		−38.3 ± 36.5			−90.8 ± 31.7	0.0068	0.0119
NPi (%)	4	0.4 ± 1.1		−3.5 ± 1.3	0.009	0.0238	−0.4 ± 1.3		
	8	−1.6 ± 1.3		−2.2 ± 1.7			−2.9 ± 1.2	0.0192	
HUS (min)	4	−14.5 ± 12.5		31.2 ± 11.2	0.0084		19.8 ± 10.0		
	8	−9.0 ± 8.3		40.9 ± 12.1	0.0018		14.9 ± 11.2		
Urine volume voided/void (mL)	4	3.6 ± 5.4		15.8 ± 4.0	0.0004		5.0 ± 4.3		
	8	1.8 ± 5.8		20.4 ± 6.1	0.002	0.0302	3.9 ± 4.4		
Urine volume voided/void in the daytime (mL)	4	3.9 ± 4.5		16.6 ± 3.3	<0.0001	0.0257	5.1 ± 2.8		
	8	4.4 ± 5.4		15.1 ± 5.1	0.005		5.0 ± 3.9		
Urine volume voided/void at night (mL)	4	−12.5 ± 16.3		28.2 ± 20.3			11.2 ± 13.4		
	8	−7.8 ± 16.6		41.6 ± 13.6	0.0041	0.0242	20.5 ± 13.0		

All values were expressed as mean ± SE

*NPi* nocturnal polyuria index (percentage of nocturnal urine volume/24-h production), *HUS* hours of undisturbed sleep (the time to the first night-time voiding), *N-QOL* Nocturia Quality-of-Life Questionnaire

Control, α-blocker alone; IM twice/day, α-blocker plus 0.1 mg imidafenacin twice daily; IM nightly, α-blocker plus 0.1 mg imidafenacin nightly

^†^Average + SE, *p* value: intragroup (baseline vs. 4 and 8 weeks); paired *t* test

^‡^Average ± SE, *p* value: intergroup (control vs. 0.1 mg imidafenacin twice/day or 0.1 mg imidafenacin nightly); ANOVA at baseline; unpaired two-sided *t* test in changes from baseline of FVC; Mann–Whitney *U* test for changes in baseline N-QOL from baseline

### N-QOL score, OABSS, IPSS and IPSS-QOL

The total N-QOL score and subdomain scores at baseline were similar among the three treatment groups (Table 1). However, a marked increase was found in the N-QOL scores of the IM twice/day and IM nightly groups at 4 and 8 weeks (Table 3). The OABSS significantly improved in all groups from the baseline to 8-week values (changes from baseline: −2.0 ± 0.4, control group; −2.9 ± 0.5, IM twice/day group; −2.2 ± 0.3, IM nightly group, not significant by Kruskal–Wallis test). Similarly, the IPSS also improved in all groups compared with the baseline values (changes from baseline: −2.4 ± 0.8 in the control group, −3.4 ± 1.1 in the IM twice/day group, and −3.6 ± 0.7 in the IM nightly group, not significant by Kruskal–Wallis test). Further, the IPSS-QOL improved in all groups compared with the baseline values (changes from baseline: −0.5 ± 0.2 in the control group; −1.3 ± 0.2 in the IM twice/day group; and −1.2 ± 0.2 in the IM nightly group, *p* = 0.013, <0.0001 and <0.0001, respectively). Compared with the control group, the other two groups showed significantly improved IPSS-QOL scores (*p* = 0.0040 and 0.0377, respectively).

### Tolerability and safety

Treatment-emergent AEs were reported by no subjects in the control group, 18.5 % (10/54) of subjects in the IM twice/day group, and 25 % (12/48) of subjects in the IM nightly group (Table 4). Imidafenacin plus α_1_-blocker treatment was not associated with an increased PVR (changes from baseline: 3.9 mL in the control group, 6.8 mL in the IM twice/day group, and 7.5 mL in the IM nightly group). There were no reports of urinary retention.

**Table 4:** Summary of treatment-emergent adverse events

	Control (*n* = 50)	IM twice/day (*n* = 54)	IM nightly (*n* = 48)
*AEs [n (%)]*			
Dry mouth	0 (0 %)	2 (3.7 %)	6 (12.5 %)
Constipation	0 (0 %)	3 (5.6 %)	1 (2.1 %)
Blurred vision	0 (0 %)	0 (0 %)	1 (2.1 %)
Anorexia	0 (0 %)	1 (1.9 %)	0 (0 %)
Insomnia	0 (0 %)	0 (0 %)	1 (2.1 %)
Palpitations	0 (0 %)	1 (1.9 %)	0 (0 %)
Oedema in the limbs	0 (0 %)	0 (0 %)	1 (2.1 %)
Gastric ulcer	0 (0 %)	1 (1.9 %)	0 (0 %)
Tremors	0 (0 %)	1 (1.9 %)	0 (0 %)
Abdominal pain	0 (0 %)	1 (1.9 %)	0 (0 %)
Pain	0 (0 %)	0 (0 %)	1 (2.1 %)
Agnail	0 (0 %)	0 (0 %)	1 (2.1 %)
Urinary AEs suggestive of AUR [*n* (%)]	0 (0 %)	0 (0 %)	0 (0 %)

Control, α-blocker alone; IM twice/day, α-blocker plus 0.1 mg imidafenacin twice daily; IM nightly, α-blocker plus 0.1 mg imidafenacin nightly; AE, adverse event; AUR, acute urinary retention

## Discussion

The Good-Night study is the first prospective randomised clinical trial that focused on nocturia, NP, and OAB symptoms, in order to evaluate the efficacy and safety of anticholinergics in male subjects on continued α_1_-blocker treatment. Additionally, this study showed the effect of anticholinergics on nocturia and NP in a real-life setting for the first time.

Nocturia has shown a poor clinical response to traditional OAB therapies, including anticholinergics [10]. Thus, other types of therapy will be needed to achieve a clinically significant reduction in nocturia. The use of the arginine–vasopressin analogue desmopressin in the treatment of NP has been studied in certain groups of patients, because it is an antidiuretic. Low doses of desmopressin administered to elderly subjects were found to reduce NP [21]. However, desmopressin is associated with the risk of hyponatremia, which is the main potentially serious associated AE, and therefore, desmopressin is currently not recommended for patients >65 years old. Considering that the prevalence of nocturia and NP increases with age, the risk of AEs associated with some of the available medications warrants careful selection to optimise the therapeutic index.

A previous study on men with nocturia found an 83 % incidence of NP; 20 % of patients had NP alone, while 63 % also had small nocturnal bladder capacity, bladder outlet obstruction, or sleep apnoea [22]. The current study seems to mirror real-life settings: 78 % of the subjects had OAB with NP. Further, the distribution of α_1_-blocker use in the treatment period, mean duration of α_1_-blocker use, and types of α_1_-blockers used before study entry did not differ significantly among the groups. Thus, the results of the current study were not influenced by the use of α_1_-blockers.

A well-known study known as the TIMES study showed the efficacy of combination therapy using α_1_-blockers and anticholinergics in male LUTS patients [13]. However, in TIMES study and other similar trials, the urine volume was not assessed. The objectives of the TIMES study thus differed from those of the Good-Night study, which focused on nocturia in male LUTS patients.

The primary endpoint in the Good-Night study, i.e. changes in night-time voiding frequency as determined using FVCs, was significantly reduced in the IM twice/day and IM nightly groups compared with that in the control group. This result is consistent with the findings of previous anticholinergic studies [13–15].

Unfortunately, in the present study, the baseline nocturia severity was lower in the control group than in the both study arms. Therefore, there may have been more room for improvement in the intervention groups than in the control group. However, no significant difference was found in the baseline OABSS among the groups, and OABSS nocturia score was similar among the groups (control group, 2.5 ± 0.5; IM twice/day, 2.7 ± 0.5; IM nightly group, 2.7. ± 0.5; *p* = 0.2290 by Kruskal–Wallis test, data not shown). Thus, it is unlikely that the differences in nocturia severity at baseline affected the results. In addition, the stratified analysis of patients with three or more nocturnal micturitions per night (baseline nocturnal micturition frequency: control group, 3.7 ± 0.3; IM twice/day, 4.4 ± 0.3; IM nightly group, 3.9 ± 0.2; *p* = 0.1459 by ANOVA) indicated that patients treated with imidafenacin showed a significantly greater reduction in the frequency of nocturnal micturition compared to the control group [mean changes ± standard error (SE) from baseline to 8 weeks: control group, −0.1 ± 0.3; IM twice/day: −1.1 ± 0.2; IM nightly group, −0.7 ± 0.3, *p* = 07671, <0.0001 and 0.0238, respectively].

The Good-Night study prospectively showed for the first time, to the best of our knowledge, that anticholinergics could reduce nocturnal urine volume in male LUTS patients with residual OAB symptoms, which is a key finding. Our data support those of previous reports: one previous study showed that imidafenacin decreased urine volume through suppression of C-fibres in the rat bladder [11], and another showed that it clinically reduced nocturnal urine volume [12], although the latter study was mainly conducted on women with incontinence and NP. Imidafenacin has a unique pharmacokinetics profile: its half-life in blood is 2.9 h (it is referred to as a short half-life anticholinergic), but its duration of receptor binding is longer in the bladder than in other organs (6–9 h in the bladder, 1–3 h in the submaxillary gland; no observation in the brain), and its metabolite has no pharmacological activity [23, 24]. These properties differ from those of other anticholinergics. A possible explanation for how imidafenacin decreases the nocturnal urine volume is that it decreases the nocturnal urine volume directly by inhibiting bladder afferent nerves and/or indirectly by improving sleep disturbance. Elderly patients treated with sleeping pills have been shown to excrete smaller amounts of urine during the night [25], thus suggesting that sleep can result in reduced urine production.

In the current study, the IM twice/day group showed prolonged HUS, reduced night-time and daytime voiding frequency, and increased urine volume voided/void, as well as a tendency towards reduced NPi. These results are consistent with previous findings [12].

Anticholinergics are commonly believed to have no effect on urine production; however, the IM nightly group showed significantly reduced urine production compared with the control group. During the period when the blood level of imidafenacin was high, urination was suppressed, particularly in the night-time, when urine production increases in patients with NP. In contrast, when the blood level of imidafenacin decreased in the daytime, urination was no longer suppressed, so the fluid balance over 24 h was normalised. Therefore, anticholinergics with a long half-life may be poor agents for reducing urine production, because their antidiuretic effect persists throughout the day. For instance, while tolterodine has shown an antidiuretic effect in rats [11], the effect of extended-release tolterodine on urine production has not been clinically proven.

Another primary endpoint was the N-QOL score. Using this score as an efficacy index, the Good-Night study found that anticholinergic add-on therapy was more effective than α_1_-blocker monotherapy in male LUTS patients with residual OAB symptoms. It clearly showed that the N-QOL scores in the IM twice/day and IM nightly groups at 4 and 8 weeks were significantly increased from baseline and that the score in the IM twice/day group, but not the IM nightly group, was significantly higher than that in the control group. This improvement in HUS with imidafenacin may have important QOL and compliance benefits.

There have been concerns that the inhibitory effect of anticholinergics on detrusor muscle contraction could theoretically aggravate voiding difficulty, increase PVR, or cause urinary retention. However, in the Good-Night study, the PVR did not significantly increase from baseline to week 8 in any of the three groups.

The main study limitation is that this is a randomised but open-label, no-placebo and non-double-blinded study. To confirm the efficacy of a particular drug, a study should determine whether it is superior to a placebo and not inferior to a pre-existing drug. However, previous phase three trials’ sub-analysis showed that imidafenacin 0.1 mg twice/day significantly reduced nocturnal frequency and nocturnal urine volume compared with placebo group [12]. Further research is required to draw a definitive conclusion regarding the reduction in nocturnal urine production by anticholinergics.

In conclusion, add-on therapy with a short half-life anticholinergic provides additional benefits for men with LUTS and nocturia who are undergoing α_1_-blocker therapy. Further, a short half-life anticholinergic may reduce nocturnal urine volume in addition to nocturnal frequency.
